# Predictors of Polypharmacy Among Elderly Patients in China: The Role of Decision Involvement, Depression, and Taking Chinese Medicine Behavior

**DOI:** 10.3389/fphar.2021.745688

**Published:** 2021-12-06

**Authors:** Chaoyi Chen, Zhanchun Feng, Qian Fu, Jia Wang, Zehao Zheng, Hao Chen, Da Feng

**Affiliations:** ^1^ School of Medicine and Health Management, Tongji Medical College of Huazhong University of Science and Technology, Wuhan, China; ^2^ School of Pharmacy, Tongji Medical College of Huazhong University of Science and Technology, Wuhan, China; ^3^ Second People’s Hospital of Yichang City, Yichang, China

**Keywords:** polypharmacy, multimorbidity, chronic diseae, multiple medication, older patient

## Abstract

**Introduction:** The prevalence of polypharmacy is gradually increasing in geriatrics, which may contribute to adverse effects, such as potential drug–drug and drug–disease interactions. These side effects remain an important challenge in patient safety, which has a significant impact on mortality and incidence rate.

**Aims:** Therefore, this study aims to understand the epidemiology of polypharmacy and identify factors that have an impact on the management of potentially inappropriate prescribing.

**Methods:** This study is a cross-sectional study, analyzing the prescription data from 720 hospitalized patients aged 50+ with a random cluster sampling method. We used inverse probability treatment weighting (IPTW) method to group and match polypharmacy and non-polypharmacy patients, and logistic regression was conducted to explore the factors associated with polypharmacy.

**Results:** The prevalence of polypharmacy accounted for 50.14% among the old patients in this study. Female patients (67.34%) have more polypharmacy than male patients, and key predictors associated with polypharmacy in the logistic regression model included the following: domicile (AOR = 0.63, 95% CI 0.42–0.95), annual income (AOR = 0.38, 95% CI 0.20–0.70), the number of chronic diseases (AOR = 3.68, 95% CI 2.69–5.06), taking Chinese medicine (AOR = 1.70, 95% CI 1.22–2.36), decision involvement (AOR = 1.49 95% CI 1.10–2.03), and depression (AOR = 1.42, 95% CI 1.03–1.96).

**Conclusion:** Polypharmacy is common among the participants with chronic diseases in Hubei province, China. The study emphasizes that gerontology practitioners should be prudent in applying clinical guidelines to provide personalized, comprehensive assessment of decision making of prescriptions, especially in socioeconomically deprived areas.

## Introduction

Multimorbidity, commonly defined as the coexistence of two or more chronic diseases in a single individual (Diederichs et al., 2011), has become a global concern following the health expectation increase among chronic disease patients. Some studies revealed that the prevalence of multimorbidity among the middle-aged and elderly people in China has ranged from 57.0 to 74.0% (Shuaishuai et al., 2021), which is higher than 59.4% in Canada (ranged from 16.9 to 59.4%) and 36.6% in European countries (Nguyen and Jeannie, 2019; Laires et al., 2020). In particular, among the older patients with multimorbidity, multiple medication regimens under the treatment of concurrent chronic diseases also increased the polypharmacy risks (Hajjar et al., 2007; Nguyen and Jeannie, 2019).

Polypharmacy is generally defined as the concurrent use of five or more medications (Lee et al., 2020). It has been widely reported that for the elderly patients there exists big health risk caused by polypharmacy (Cadogan et al., 2016). For example, due to the frail elderly patient’s declined renal and hepatic function with long-term use of multiple medicines, they cannot be metabolized in their body, which may cause further damage to their organs (Venturini et al., 2011; Masnoon et al., 2017; Wastesson et al., 2018). Polypharmacy was linked to adverse events and poor health outcomes including falls, adverse drug effects, even increasing the rate of hospital admission, and mortality (Scott et al., 2015; Masnoon et al., 2017; Wastesson et al., 2018).

Seriously, patient safety is one of the most crucial targets of the health system, which is essential to achieve Universal Health Coverage (UHC) (Organization, 2019). However, polypharmacy is a typical and widespread public health problem among the older population in China (Lai et al., 2018). Therefore, well-understood epidemic characteristics of polypharmacy and identifying the impact factors of how physicians and elder patients manage their potentially inappropriate prescription behaviors were necessary. Recently, many pieces of evidence confirmed that polypharmacy is associated with basic demographic characteristics, comorbidity, multiple specialist diagnosis, and patients’ self-medication knowledge and driven by a lower level of shared decision-making behaviors (Halli-Tierney et al., 2019; Khezrian et al., 2020; Liau et al., 2021), but the fields of polypharmacy and its relationship with taking Chinese medicine behavior and depression symptoms require in-depth research.

To address this gap, we conducted this study by the inverse probability treatment weighting method, which could be used to reflect the polypharmacy status and update insights on the prevalence of multiple medications in China, and then explored the factors influencing patients’ polypharmacy. Meanwhile, the evidenced strategies could be provided to improve the elderly patients’ rational drug use and their health outcomes.

## Methods

### Participants and Procedure

This study was conducted from March to May 2021. We first selected eight administrative regions (including Jianghan District, Jiang’an District, Qiaokou District, Hongshan District, Wuchang District, Hanyang District, Caidian District, and Jiangxia District) from 13 administrative regions in Wuhan, Hubei province, China, and then randomly selected from the administrative regions in eight tertiary hospitals. Patients (≥age 18) having at least one chronic disease (such as hypertension, heart disease, and diabetes) and routine daily medication for 3 months or more were recruited to participate in the survey. Potential participants were invited by trained investigators. Before beginning the investigation, each patient needs to fill in an informed consent or orally agree to participate in the survey.

## Measures

### Dependent Variable

We assessed polypharmacy medication by using this single question: how many kinds of drugs have you taken to treat your chronic diseases in the last 3 months? According to previous studies consider taking of 5 or more drugs at the same time to be multi-drugs (Charlesworth et al., 2015; Onder et al., 2005). This study regards taking five or more drugs simultaneously as polypharmacy. In our study, we divided this behavior into two categories: taking 0–4 drugs is regarded as non-polypharmacy, while taking five or more drugs is interpreted as polypharmacy.

### Instrument Development

To explore the factors that influenced the patients’ polypharmacy and their participation in the medication decision-making process, we designed a self-developed survey tool, which included four parts: the basic demographic information (age, domicile, gender, and income), treatment decision involvement of patients, risk perception, self-care-related health information, and emotional status.

### Response Variables

1) Decision involvement. The shared decision-making tool (SDM-Q-9) (Kriston et al., 2010) mainly includes three dimensions (nine measurement items): 1) information exchanges (the doctor communicated with me about the medication regimen; the doctor talked with me about which medication treatment is more appropriate; I had plenty of time to communicate with the doctor); 2) participation (in the selection of medication, the doctor ever asked my advice; I asked the doctor about the pros and cons; the doctor encouraged me to participate in the choice of the medication regimen); 3) reaching an agreement (I weighed the pros and cons of different medication regimens with professionals finally; I made the final medication treatment decision together with the doctor; I agreed with the doctor on which medication regimen to use). Responses are provided with a 5-item Likert scale, from 1 (completely disagree) to 5 (completely agree), and the total score is 45. This section was divided into two grades: 1) the total score is ≥29 (average score), regarded as high decision involvement; 2) the total score is less than the average score, which is perceived to be at a low level. In addition, the Cronbach’s alpha coefficient was 0.876, which means that it has high reliability. To assess the instrument’s validity, average variance extracted validity (AVE) is used (Table 1). Its value is 0.613, which is greater than 0.5, indicating that the SDM tool has good validity (Yang and Zhang, 2014).
2) Depression scale. This dimension was measured by the 10-item Center for Epidemiological Studies-Depression Scale (CES-D10) (Verger et al., 2009). The answers for CES-D10 are on a four-scale metrics coding from 0 to 3 (0 = less than 1 day; 1 = 1, 2 days; 2 = 3, 4 days; 3 = 5–7 days). The total score of the scale ranged from 0 to 30, with the higher score indicating more depressive symptoms, and CES-D10 has been used in previous studies and it showed good internal reliability and validity (Andresen et al., 1994).
3) Risk perception. It comprises 10 items for evaluating the individual’s perspective of economic burden risk, psychology risk, health risk, and time risk with a 5-item Likert scale from 1 (totally disagree) to 5 (totally agree) during medication. The standardized Cronbach’s *α* coefficient was 0.785, and AVE was 0.453 (Table 1).
4) Health related items. They include the number of chronic diseases, whether taking traditional Chinese medicine or not, and have you ever had any adverse drug reactions during the medication?

**TABLE 1:** Reliability and validity of the survey instrument.

Variables	Cronbach’s alpha	Composite reliability	Average variance extracted
Decision involvement	0.876	0.904	0.613
CES-D10	0.750	0.818	0.517
Risk perception	0.785	0.786	0.453

### Statistical Analysis

Descriptive statistics were reported as frequency and percentage. The patients with chronic diseases of polypharmacy were regarded as the treatment group, and the patients of non-polypharmacy were regarded as the control group. χ^2^ tests were used to examine the factors associated with polypharmacy. For retaining the sample complete information and controlling the bias of the estimation results caused by the selection bias and endogenous problems, we used an inverse probability treatment weighting (IPTW) method to group and match dependent variables, and then a balance weighted test of covariates was conducted by verifying the matching effect, and propensity value weighted regression analysis was carried out to further predict the impact of the vital factors and pathway on the polypharmacy of the elderly patients. In this study, *p*-values of <0.05 were considered to be statistically significant. Statistical analyses and plot forest were performed by R3.6.0 software and Graph-Pad Prism 9.0.

## Results

### Characteristics of the Study Population

A total of 720 respondents participated in this study, and 536 people were ≥60 years old, accounting for 74.45%. In the sample, the average age was 73.56 years (ranging from 50 to 101 years), 42.08% (n = 303) were male, 77.30% resided in urban cities, 179 people were reported to live alone, 57.92% (n = 417) have obtained junior high school and below degrees, 63.94% reported annual individual income ≥37,600 Chinese Yuan (CNY), and 72.50% had urban medical insurance (Table 2).

**TABLE 2:** Descriptive characteristics of the study population.

Characteristics	Respondents (N = 720)	Proportion (%)
Gender		
Male	303	42.08
Female	417	57.92
Age		
<60	184	25.55
60–70	284	39.45
>70	252	35.00
Education		
Junior high school and below	417	57.92
High school	188	26.11
College and above	115	15.97
Domicile		
City	556	77.30
Rural	164	22.70
Living status		
Alone	179	24.86
Not alone	541	75.14
Annual individual income		
<16,400 Yuan	80	11.11
16,400–28,399 Yuan	63	8.70
28,400–37,599 Yuan	117	16.25
≥37,600 Yuan	460	63.94
Medical insurance		
Purchased	522	72.50
None	198	27.50

### Polypharmacy Among the Elderly Patients

Overall, the results show that 361 people took five or more drugs (polypharmacy), accounting for 50.14%, and 359 have been identified as non-polypharmacy. This study found a significantly higher percentage of polypharmacy among the older adults who were suffering from three or more chronic diseases (69.53%); in addition, female patients (N = 207, 57.34%) have more multiple medications than male patients (N = 154, 42.66%), and urban residents (N = 292, 80.89%) had a higher prevalence of polypharmacy. Furthermore, the individuals who took Chinese medicine (52.35%) recently and who showed higher levels of depression (54.85%) tend to take multiple medications (Table 3).

**TABLE 3:** Characteristics of polypharmacy and non-polypharmacy among the participants.

	Polypharmacy		Non-polypharmacy		
Characteristic	Frequency	Percentage (%)	Frequency	Percentage (%)	*p*
Age (years)					0.638
<60	87	24.10	97	27.02	
60–70	147	40.72	137	38.16	
>70	127	35.18	125	34.82	
Domicile					0.029
Urban	292	80.89	264	73.54	
Rural	69	19.11	95	26.46	
Gender					0.753
Male	154	42.66	148	41.23	
Female	207	57.34	211	58.77	
Level of education					
Junior high school and below	201	55.68	215	59.89	0.435
High school	102	28.25	87	24.23	
College and above	58	16.07	57	15.88	
Annual Individual income/Yuan					
<16,400 Yuan	42	11.63	38	10.58	
16,400–28399Yuan	31	8.59	32	8.91	
28,400–37,599 Yuan	42	11.63	75	20.89	0.008
≥37,600 Yuan	246	68.14	214	59.61	
Medical insurance for urban residents					0.838
None	101	27.98	97	27.02	
Have	260	72.02	262	72.98	
Living status					0.131
No	80	22.16	98	27.30	
Yes	281	77.84	261	72.70	
Number of diseases					0.013
0–2	110	30.47	215	59.89	
>3	251	69.53	144	40.11	
Medical institution visited					
Primary medical institution	21	5.82	24	6.69	0.171
Non-primary medical institution	298	82.55	277	77.16	
Uncertain medical institution	42	11.63	58	16.16	
Risk perception					0.944
High	191	52.91	188	52.37	
Low	170	47.09	171	47.63	
Depression					0.001
High	198	54.85	150	41.78	
Low	163	45.15	209	58.22	
Adverse drug reaction					0.361
No	74	20.50	63	17.55	
Yes	287	79.50	296	82.45	
Decision involvement					0.020
High	180	49.87	148	41.23	
Low	181	50.13	211	58.77	
Taking Chinese medicine					0.000
No	172	47.65	239	66.57	
Yes	189	52.35	120	33.43	

### Logistic Regression Analysis Results Before Propensity Score Weight Matching

**FIGURE 1:**
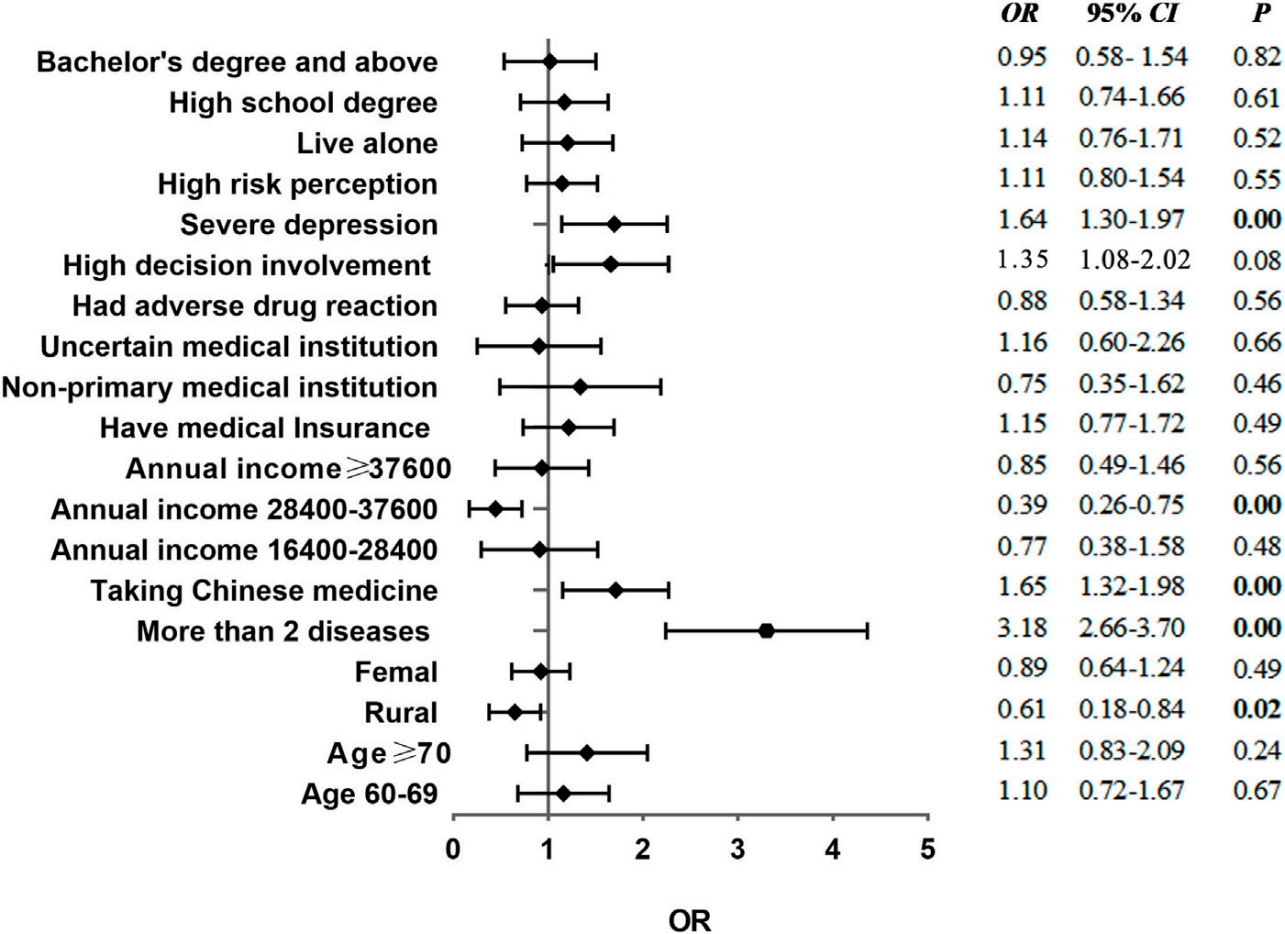
Forest plot of logistic regression analysis results of polypharmacy before matching.

The adjusted OR (AOR) and 95% CI from binary logistic regression analysis (before matching) are displayed in Figure 1. The results showed that respondents who have more than two chronic diseases (OR = 3.18, 95% CI = 2.66–3.70) and rural households (OR = 0.61, 95% CI = 0.18–0.84) are less prone to polypharmacy than the urban. Patients who take traditional Chinese medicine (OR = 1.65, 95% CI = 1.32–1.98) are more likely to exhibit polypharmacy than those who do not take. In addition, patients with the annual income in the second interval of 28,400–37,600 Yuan (OR = 0.39, 95% CI = 0.26–0.75) had a significant association with polypharmacy. Patients with severe depression (OR = 1.64, 95%CI = 1.30–1.97) are more likely to have multiple medications.

### Logistic Regression Analysis Results After Propensity Score Weight Matching

**FIGURE 2:**
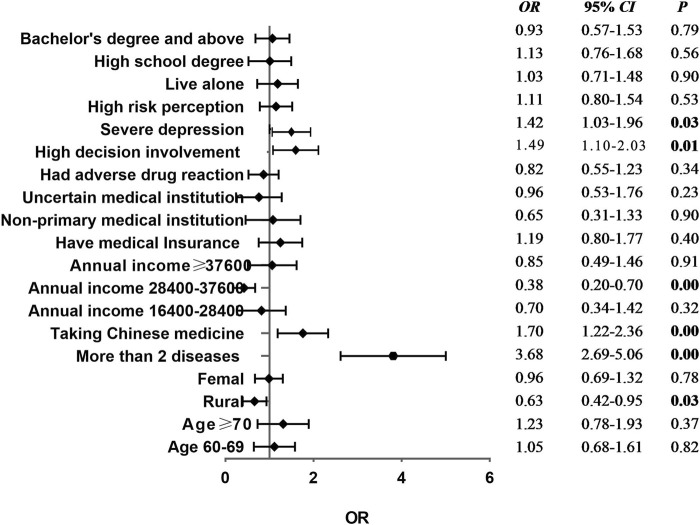
Forest plot of logistic regression analysis results of polypharmacy after matching.

By using the propensity score weighted (PSW) matching method for adjusted effect evaluation in the logistic regression analysis results of polypharmacy, it was found that the regression coefficient of the model increased from 0.46 to 0.63, which indicated that the predictive effect of the pair was enhanced. Figure 2 shows that adjusted OR results and relationship between decision-making involvement, the type of domicile, whether to take traditional Chinese medicine, annual income, depression degree, and the number of diseases were remaining statistically significant (*p* < 0.05), compared with the unmatched results. After verification and analysis, it indicated this empirical model has a certain degree of robustness.

## Discussion

This study was performed to describe the prevalence of polypharmacy among the elderly patients in China. We found that the rate of polypharmacy was high as nearly 50.14% elder patients with chronic diseases were prescribed five or more medications. Similar rates (44.90–83.50%) were reported in the previous literature (Chan et al., 2009; Kim et al., 2014). As expected, the likelihood of polypharmacy was correlated with main factors: the type of domicile, annual individual income, the number of chronic diseases, taking Chinese medicine behavior, depression symptoms, and decision involvement.

In the cohort of patients who were reported to live in rural areas (AOR = 0.63, 95% CI 0.42–0.95), there was weakening of the association with polypharmacy. It is likely that they have poor access and availability of various chronic medicines in the region they live. As community-level medical institutions can only provide essential medicines in the township (Rixiang et al., 2018; Yuanzheng, 2018), to some degree, the range of medicines supplied by primary medical institutions was limited. It can discount excessive medicine usage. In addition, compared with urban residents, the rural have a lower household income, which cannot afford redundant medical and health expenditure; as a result, they rely on several common medications (nifedipine, simvastatin, and metformin) for general therapy of hypertension and diabetes. Therefore, the probability of combination of other medications in this group is relatively small.

Compared with the low-income group, the middle-income group (28,400–37,600 Yuan) was more inclined to go to primary-level medical institutions for medical treatment (Hongme et al., 2020). The physicians of the community health service institute as the “public health gatekeeper” only undertake the function of diagnosis and treat symptoms, but if a large number of drugs are prescribed to the patients in the local area, it cannot be accepted by the local residents. Conversely, high-income groups are more willing to use health services in municipal/provincial general hospitals with higher convenience and accessibility. In this clinical scenario, they could approach more medicines from multiple prescribers, which increase the risk of multiple medication use. Furthermore, it leads to a prolonged hospital stay in which the “prescribing cascades” are identified and corrected (Schenker et al., 2019).

The proportion of elderly patients taking traditional Chinese medicine was high, and a previous study highlighted the potentially high impact of traditional Chinese medicine on polypharmacy in Chinese populations (Chan et al., 2015). This is in line with our study; the participants who tend to take traditional Chinese medicine were more likely to take multiple medications (Lai et al., 2018). One explanation was based on the construal level theory (McCrea et al., 2012; Lermer et al., 2016); the lower-level construal group indicated that the elderly people with health problems mainly focus on immediate goals and not the long-term health needs. Under these circumstances, obtaining traditional Chinese medicine will become a process to fill the psychological gap. It simply proposes that the psychological distance of medication behavior decision making is acceptable compared with physical examination and hospitalization. Besides, according to previous interviews with the respondents in this survey, we noticed that for the people who tend to take Chinese medicine, it may be due to their low medical knowledge literacy and insufficient information about adverse outcomes and harm of various medicines, and they mistakenly believe that the direct way to control the disease is to take different kinds of medicines to treat the disease, which result in polypharmacy correspondingly.

We found that polypharmacy is mostly a consequence of multiple chronic diseases; this is consistent with studies from Mina Khezrian and Yuxin Liu, and coexistence of multiple chronic diseases is prevalent in frail people, resulting in a decline in the cognitive status and increased probability of taking multiple medications (Khezrian et al., 2020; Zhang et al., 2020; Liu et al., 2021). Another explanation is that the physicians need to make complicated and long-term therapy to achieve the desired health outcomes for individual patients (Ellis et al., 2020). Thereby, more medication regimens were used in treatment, which would also cause prescribing cascades (Alwhaibi et al., 2018). Furthermore, the residents of the survey area have a low level of health information literacy (Shilong et al., 2015); they could ignore and underestimate the potential health risks of polypharmacy.

Noteworthy surveys have proved that depression was a significant independent predictive factor for polypharmacy in elderly (Marengoni et al., 2011; Yavuzer et al., 2017), and we also found that people who were reported to have high depression symptoms were more likely to exhibit polypharmacy. Psychological problems would increase the general susceptibility of having functional disability or cognitive impairment (Burnier et al., 2020). In addition, it distressed patients’ adherence to drug therapy, which caused polypharmacy for reducing their self-concerns about health problems. In the absence of practice guidelines and external medication supervision, taking a large number of medications was regarded as the psychological protection of chronic physical disorders (Smith et al., 2014). Therefore, severe depression may contribute to excessive polypharmacy.

Patients who tend to make joint decisions between physicians and patients are less likely to take multiple medications; increasing clinical evidence indicates that patients’ involvement in medical decision making improves health care outcomes (Alden et al., 2014). In short, if a patient has been fully involved in the decision as an equal collaborator, they can understand the critical issues and share information provided by physicians and make a rational treatment choice (Rostoft et al., 2020). Before finalizing the medication regimen, clinicians should balance the benefits and risks with polypharmacy (Zhang et al., 2020). Indeed, appropriate communication of medicine regimens contributes to preventing polypharmacy and negative health outcomes among frail patients (Kutner et al., 2015; Wastesson et al., 2018; Ozavci et al., 2020).

## Conclusion

Polypharmacy is common among the participants with chronic diseases in Hubei province, China. Given that several factors influencing multi-medication use were identified in this study, we suggest that health care professionals should broaden the knowledge of rational medication and improve the residents’ medication literacy. Besides, in clinical practice, physicians should be prudent in applying clinical guidelines and encourage patients to participate in decision making of prescriptions and reduce patients’ internal psychological burden. In the community, family doctors’ monitoring and assessment of patients’ use of medication have a significant impact on appropriate adherence to their prescribed drug regime.

### Limitations

Our study has several limitations. Various definitions of polypharmacy existed in the literature, and we only considered the number of drugs used, namely, ≥5 drugs as polypharmacy, so it is difficult to make a distinction between the necessary prescribing and polypharmacy medication. Second, we used the CES-D10 scale in this study, which could only screen for the presence of depressive symptoms or negative emotion. A complete diagnostic assessment of clinical depression would be conducted in the future. Third, we did not put the medication duration and medication adherence factors into our design section for providing a valuable tool, and we will continue to improve the questionnaire in subsequent research.

## Data Availability

The data analyzed in this study is subject to the following licenses/restriction: The datasets used and/or analyzed during the current study are available from the corresponding author on reasonable request. Requests to access these datasets should be directed to fengda@hust.edu.cn.

## Abbreviations

AVE, average variance extracted; CESD, Center for Epidemiological Studies-Depression Scale; CI, confidence interval; CNY, Chinese Yuan; OR, odds ratio.
